# Focused Ultrasound and Microbubbles-Mediated Drug Delivery to Brain Tumor

**DOI:** 10.3390/pharmaceutics13010015

**Published:** 2020-12-24

**Authors:** Sheng-Kai Wu, Chia-Lin Tsai, Yuexi Huang, Kullervo Hynynen

**Affiliations:** 1 Physical Sciences Platform, Sunnybrook Research Institute, Toronto, ON M4N 3M5, Canada; realismjames@gmail.com (S.-K.W.); jialin0912@gmail.com (C.-L.T.); yuexi.huang@sunnybrook.ca (Y.H.); 2 Department of Medical Biophysics, University of Toronto, Toronto, ON M5G 1L7, Canada; 3 Department of Neurology, Tri-Service General Hospital, National Defense Medical Center, Taipei 11490, Taiwan; 4 Institute of Biomaterials and Biomedical Engineering, University of Toronto, Toronto, ON M5S 3G9, Canada

**Keywords:** blood–brain barrier (BBB), blood–brain–tumor barrier (BBTB), central nervous system (CNS), convention-enhanced delivery (CED), focused ultrasound (FUS)

## Abstract

The presence of blood–brain barrier (BBB) and/or blood–brain–tumor barriers (BBTB) is one of the main obstacles to effectively deliver therapeutics to our central nervous system (CNS); hence, the outcomes following treatment of malignant brain tumors remain unsatisfactory. Although some approaches regarding BBB disruption or drug modifications have been explored, none of them reach the criteria of success. Convention-enhanced delivery (CED) directly infuses drugs to the brain tumor and surrounding tumor infiltrating area over a long period of time using special catheters. Focused ultrasound (FUS) now provides a non-invasive method to achieve this goal via combining with systemically circulating microbubbles to locally enhance the vascular permeability. In this review, different approaches of delivering therapeutic agents to the brain tumors will be discussed as well as the characterization of BBB and BBTB. We also highlight the mechanism of FUS-induced BBB modulation and the current progress of this technology in both pre-clinical and clinical studies.

## 1. Blood–Brain Barrier (BBB)/Blood–Brain–Tumor Barrier (BBTB) Basics

### Limitation of the BBB for Pharmaceutical Treatment of Diseases

The blood–brain barrier (BBB) is a cellular barrier that maintains the homeostasis of the neural microenvironment for adequate synaptic transmission [1]. The main BBB component is the brain capillary endothelial cells (ECs), and that in concert with the surrounding cells, such as pericytes, astrocytes, microglia, neurons, and the extracellular matrix, construct the neurovascular unit (NVU) [2,3]. The tight junctions (TJs) between brain capillary ECs extensively limit molecules to brain entrance across the paracellular path, except for some extremely small or gaseous molecules as well as tiny highly liposoluble molecules [4]. Additionally, brain capillary ECs display transcytosis at extraordinarily low rates as compared with peripheral ECs, which significantly restrains the vesicle-mediated transcellular transport of macromolecules [5]. The paracellular and transcellular barrier properties of BBB also set up challenges for drug delivery to the central nervous system (CNS).

There are two main types of transporters expressed in brain capillary ECs. The first is highly specific transporters that regulate the uptake of essential nutrients across the BBB. Many of these transporters belong to the solute carrier (SLC) superfamily [6,7] The second is efflux transporters, which convey a broad range of lipophilic molecules, that could passively diffuse through the cell membrane of ECs, back to the blood, and thus perform as ‘gatekeepers’ to control the brain entry of these molecules. A variety of adenosine triphosphate (ATP) binding cassette efflux transporters, such as P-glycoprotein (P-gp; also known as ABCB1), breast cancer resistance protein (BCRP; also known as ABCG2), and multidrug resistance-associated proteins (MRPs; also known as ABCCs), apply the energy of ATP hydrolysis to transport their substrates versus their concentration gradient [8]. Hence, active ATP binding cassette (ABC) efflux transporters contribute to reduce or prohibit the neurotoxic side effects of drugs. Therefore, a wide range of anti-tumor agents is incapable to cross the BBB via transcellular diffusion owing to the presence of active efflux transporters (AETs), which bestows chemo-resistance to brain tumors [9].

Impaired BBB functions can be observed in various neurological disorders such as stroke, multiple sclerosis, Alzheimer’s disease (AD), epilepsy, and brain tumors [10]. In brain tumors, the BBB is ruined with tumor progression, producing a vasculature referred to as the blood–brain tumor barrier (BBTB). The characteristics of BBTB include heterogenous pericyte coverage [11] and shrinkage of astrocyte endfeet, in which invading glioma cells can physically replace brain capillary ECs [12]. Consequently, it would lessen the barrier functions of the CNS NVU. The expression of vascular endothelial growth factor (VEGF), a potent inducer of vascular permeability, has been found to correlate with capillary permeability but not be elevated in low-grade gliomas [13]. In high-grade gliomas, the increased metabolic demands provoke hypoxic zones that stimulate enhanced expression of VEGF and neovascularization, leading to abnormal vasculature and increased vascular permeability [14,15]. Physical disruption of the BBTB is evident in the increased contrast agent accumulation within the extravascular extracellular space demonstrated by dynamic imaging acquisitions [16]. However, the enhanced permeability of BBTB is heterogeneous and does not include the advancing tumor boundary. Despite many ongoing efforts to develop novel antineoplastic agents to treat glioblastoma multiforme (GBM), none of them have demonstrated a satisfactory therapeutic efficacy [17], most likely due to sub-lethal drug concentrations in the non-enhancing parts of the tumor. Therefore, the development of methods to improve drug delivery has become a significant issue in brain tumor therapy.

## 2. Strategies Overcoming BBB/BBTB

Several studies have investigated approaches such as direct surgical injection, intranasal delivery, as well as AET- and TJ-targeted strategies to overcome and modulate the entire BBB/BBTB system in brain tumor therapy. Table 1 summarizes the strategies overcoming BBB/BBTB.

### 2.1. Direct Surgical Injection

Direct infusion of the drug via a cannula to the brain parenchyma is a straightforward strategy to bypass the BBB/BBTB and designate the bulk convective flow of the drug in the interstitial space. However, the rate of injection and the diffusion of the drug have an impact on the drug distribution [18]. The improvement in survival using this method remains insufficient in clinical trials of patients with glioblastoma [19]. Convection-enhanced delivery (CED), a more promising technique, works through applying a pressure gradient at the tip of an infusion catheter to drive therapeutics throughout the brain tumor, such that convective forces influence over diffusive transport. Recent preclinical studies and clinical trials utilizing CED in glioma models have explored a board range of potential therapeutic drugs, such as carboplatin [20] and paclitaxel [21]. Nevertheless, the results still could not confirm its efficacy due to inadequate drug penetration and accumulation in the targeted tumor region [22]. 

Gliadel wafer is another direct local delivery method for the treatment of glioblastoma, which involved controlled release delivery of the chemotherapeutic agent using a biodegradable polymer loaded with the agent and implanted into the tumor cavity after surgical removal of the tumor [23]. Therapeutic molecules incorporated in biodegradable polymers are released by the cooperation of diffusion and polymer degradation. Biodegradable wafers releasing Carmustine offered further survival improvement for patients with high-grade glioma [24] but were also associated with high complication rates [25]. 

Microchip (microelectromechanical device) technology for local chemotherapeutic delivery can provide even more control over the drug release. Recently, preclinical studies applying injectable gel-like lipid capsules and microchips have demonstrated promising results [26,27]. However, the limitations of these approaches include the necessity of invasive placement, increased risk of local neuronal injuries along the track, and advised effects such as edema, bleeding, and infections [28,29].

### 2.2. Intranasal Delivery

The intranasal delivery of the drugs is a non-invasive substitute way for targeting the CNS to bypass the BBB/BBTB and decrease the systemic adverse effects. Various therapeutic agents, such as small organic molecules, biotech complexes, and stem cells, are under investigation for GBM treatment via nose-to-brain delivery. Also, several nanostructured and nano-sized carrier systems were designed for enhancing intranasal delivery [30]. However, the majority of studies at present are in the pre-clinical phase of development, where favorable results remain under animal models. Long-term clinical studies investigating perillyl alcohol, a monoterpenoid compound derived from the essential oils of botanicals and prone to interact with the lipid bilayers of gliomas [31], showed promising results only in selected GBM patients while using intranasal delivery [32,33].

### 2.3. AET-Targeted Strategies 

AET-targeted strategies refer to circumventing active efflux across the BBB/BBTB via biochemical modifications of existing drugs as well as pharmacological inhibition of AETs. The ECs at BBB/BBTB express a range of different receptor-mediated transport (RMT) systems, such as the transferrin receptor, that are involved in the uptake of required nutrients [7]. The modifications could achieve conversion from the drug to an analog of the ligand against the specific receptor at the BBB/BBTB or link the drug to the ligand of the RMT [34,35]. Currently, the modification of drug candidates has targeted reducing their molecular size and enhancing their lipophilicity to increase their BBB/BBTB permeability. ABCB1 (P-gp) and ABCG2 (BCRP) are the two major substrates of molecularly targeted agents to diminish their affinity to AETs, enhancing drug penetration through the BBB/BBTB [36]. 

Pharmacological inhibitors of AETs are a direct approach to overcome the active efflux of anti-tumor agents. Elacridar and tariquidar are currently the two most promising options [37]. Nevertheless, studies of direct inhibition of AETs at the BBB/BBTB in vivo showed insufficient efficacy and intolerable toxicities [38], which are associated with their inadequate binding affinities [39]. Therefore, the development of an inhibitor with high specificity to AETs and suitable tolerability is warranted to overcome the active efflux of drugs in the future.

### 2.4. TJ-Targeted Strategies

TJ-targeted strategies include chemical-mediated and hyper-osmotic BBB/BBTB disruption. Chemical-mediated disruption involves the application of a specific agent that temporarily changes the BBB/BBTB integrity. A panel of vasoactive compounds has been explored, including tumor necrosis factor α (TNF-α)/interferon γ (INF-γ), alkylglycerols, bradykinin, and histamine, but evidence concerning its efficiency is limited [17]. Hyper-osmotic BBB/BBTB disruption refers to the intra-arterial infusion of the hyperosmotic solution, most frequently mannitol, leading to transient endothelial cells’ shrinkage with the subsequent opening of TJs and an elevated BBB permeability for some time [40,41]. This time window offers the chance of intra-arterial delivery of chemotherapeutics such as methotrexate and carboplatin [42]. However, invasive carotid artery injections are required. Meanwhile, the non-selective permeabilization of not only the BBTB but also the BBB allows a widespread entry of chemotherapeutic agents, causing the possibility of neurological complications such as aphasia and hemiparesis [43].

### 2.5. Focused Ultrasound-Induced Brain Vascular Permeability Increment

The BBB prevents about 98% of small molecule drugs from entering the brain parenchyma [44]; however, focused ultrasound (FUS) combined with intravenous microbubbles (MBs) has shown a promising potential of temporarily and reversibly increasing BBB permeability in a non-invasive manner [45]. Moreover, BBB permeability is restored to baseline within 6 to 24 h [46]. Therefore, this technology allows therapeutics to accumulate in sonicated regions in a desired time window. Currently, FUS can be utilized in conjunction with magnetic resonance imaging (MRI), which is able to precisely localize the pathological targets. The other invasive option is to surgically implant the transducer in a hole in the skull under the skin such that the ultrasound beam is aimed at the tumor site [47]. The transducer is activated by placing a needle electrode through the skin to the transducer to connect it to the driving electronics.

**Figure 1:**
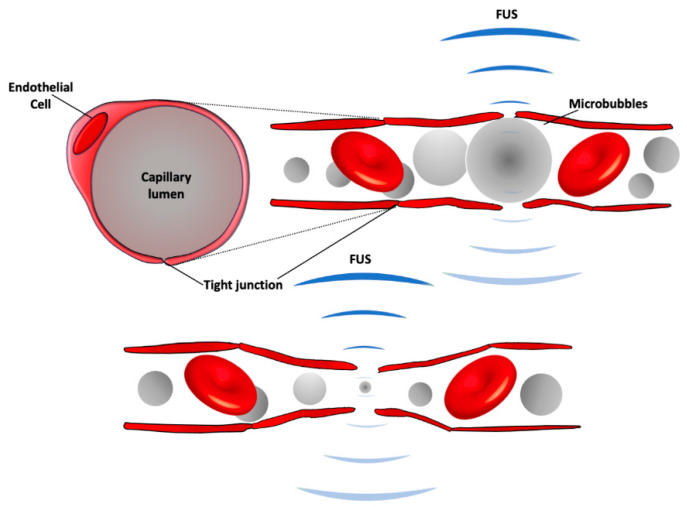
A schematic of BBB modulation following focused ultrasound (FUS) with intravenous injection of microbubbles. Circulating microbubbles expand (top) and contract (bottom) in the acoustic field, stretching the capillary walls and producing forces on endothelial cells to generate bioeffects that increase BBB permeability. The expansion and contraction happen at the frequency of the ultrasound field, which is 220,000 times/s with the clinical system.

The physical mechanism behind FUS-induced BBB modulation is known as the cavitation effects from the circulating MBs interacting with FUS sonication. Acoustic cavitation results in BBB modulation via stable or inertial cavitation. Stable cavitation contributes to tight junction modulation [48], whereas inertial cavitation is related to vessel rupture and certain degrees of hemorrhage [49]. Stable cavitation can be characterized by the emissions of harmonic signals from the bubble. With increasing pressure amplitude, sub-harmonic and/or ultra-harmonic signals appear and can be used as a calibration point for maintaining disciplinary contraction and expansion of microbubbles [50] with desired BBB permeability increase without permanent tissue damage. Broadband emissions usually relates to microbubble collapse [51], which produces microstreaming and micro-jets to the surrounding tissues and results in vascular rupture and permanent tissue damage. The physical stresses generated by interaction between ultrasound and microbubbles cause biological changes of the BBB (Figure 1). The possible routes across the BBB, including increased endocytosis/transcytosis, paracellular passage through modified tight junctions, and through the endothelium cell membrane channels, have been studied with acoustic conditions that induce transient BBB permeability enhancement [52]. The changes in tight junctions have been shown at the cellular level after MBs/FUS-induced BBB modulation using an electron microscope [53]. 

The use of FUS-facilitated drug delivery to the brain by transiently increasing the permeability of the BBB is somewhat risky. Like any medical intervention, adverse effects are always the priority to deal with. Although FUS-induced BBB modulation appears promising, FUS parameters must be properly controlled to avoid side effects such as a massive erythrocyte extravasation [49,54]. There is a delicate window of acoustic parameters as well as the use of microbubbles (MBs) that shows the feasibility of achieving the designed vascular permeability enhancement while minimizing the irreversible damages in the brain tissues. Besides the pronounced increase in vascular permeability, changes in vascular function and brain behavior should also be evaluated in FUS + MBs treatment. 

In this section, ultrasound parameters that affect the degree of therapeutic outcomes will be discussed. Inflammation, which is a natural response to injury upon restoring homeostasis, can be generally classified as acute and chronic. The inflammation in the CNS is a double-edged response that might help with recovery from the injury, however might simultaneously worsen pathology [55]. The BBB is essential in the maintenance of the brain microenvironment by restricting the passage of harmful and pro-inflammatory substances such as albumin and fibrinogen from the systemic circulation. Except for severe damages regarding the undesirable control of FUS parameters, FUS-induced BBB modulation under a proper feedback control causes some acute inflammatory responses and has drawn much more attention recently [56]. However, there are variations in the published data of this topic in regard to acoustic exposures [57,58,59]. Transcriptome analysis showed an acute increase in proinflammatory cytokine genes in 6 h following sonication, whereas the expressed genes return to their baseline mainly by 24 h [60]. A sterile inflammatory response, including the increase in cytokines and cell adhesion molecules, following FUS-induced BBB modulation was investigated in one study [57], while the response persisted less than 24 h and transcriptional changes and microglia/astrocyte activation restored in approximately 2 weeks [60]. In addition, FUS-induced BBB modulation could also facilitate brain vascular angiogenesis as well as immune recognition with the aid of interleukin-12 (IL-12) [61,62]. In brief, the microenvironment changes via FUS-induced BBB modulation might be beneficial if the induced neuroinflammation can be regulated and reversible.

**Figure 2:**
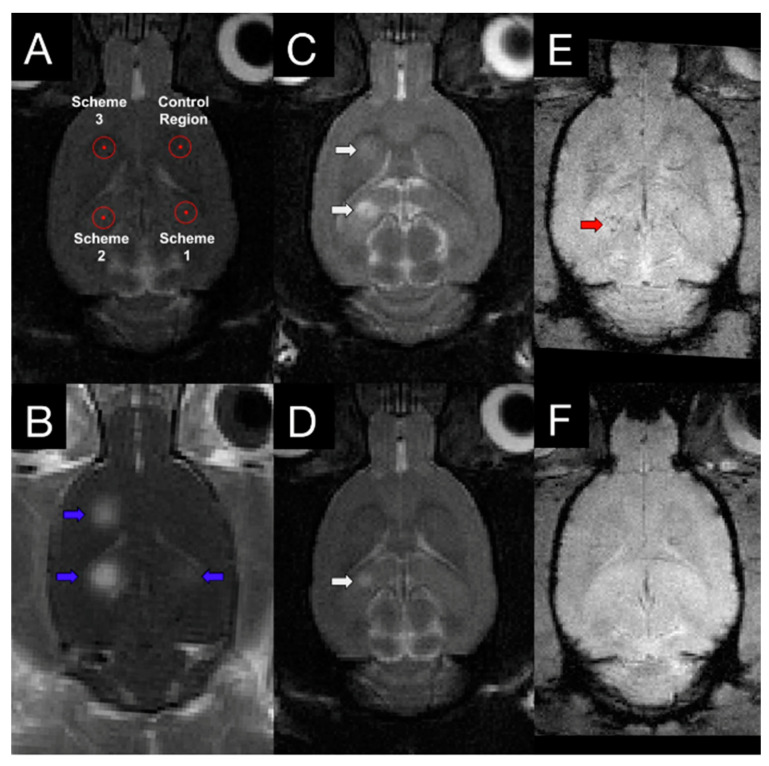
T1w, T2w, and T2*w images following FUS treatment. (**A**) Three sonication targets with different FUS parameters (scheme 1: acoustic controller was used with 10 μL/kg microbubbles; scheme 2: fixed 0.29 MPa was used with 100 μL/kg microbubbles; scheme 3: acoustic controller was used with 100 μL/kg microbubbles) and one control area were chosen based on T2w image. (**B**) Contrast-enhanced T1w image showed increased BBB modulation region, indicating with blue arrows. T2w images at 4 h (**C**) and 4 days (**D**) following FUS treatment demonstrated different degrees of edema at schemes 2 and 3 (white arrows). T2* images were also acquired at 4 h (**E**) and 4 days (**F**) post-sonication. Hypointensity in T2* (red arrows) was observed in one animal at scheme 2. With the proper control of FUS parameters, a safe and reversible BBB permeability modulation could be achieved. Adapted from [56], Theranostics, 2017.

The efficacy and safety of FUS + MB treatments have been assessed by different measures evaluating the degree and duration in the altered BBB permeability. Magnetic resonance imaging (MRI) can serve as a tool to evaluate the effects MBs/FUS-induced in the central nervous system (CNS). T1-weighted image is one of the basic pulse sequences in MRI and demonstrates differences in the T1 relaxation times of tissues. Since T1-weighted (T1w) sequences provide the best contrast for paramagnetic contrast agents (e.g., gadolinium-containing compounds), BBB permeability change following MBs/FUS treatment can be evaluated by contrast-enhanced T1-weighted (CE-T1w) images. With the effect of reducing T1 relaxation time and thereby increasing the signal intensity, the hyperintensities in targeted areas indicate increased BBB permeability. The dynamic contrast-enhanced MRI (DCE-MRI) technique for kinetic analysis of delivered molecules is capable of describing FUS-induced BBB modulation in a semi-quantitative measure [63,64,65]. The transfer coefficient, K_trans_, defined as the influx volume transfer constant from plasma to extracellular extravascular space in CNS tissues, has been widely used as an index to describe FUS-BBB modulation in DCE-MRI kinetic modeling. In addition to detecting the permeability enhancement, CE-T1w images can also be a tool to investigate the BBB reversibility after FUS. BBB permeability restores within 6–24 h and the time required for restoration to baseline following FUS-BBB modulation is independent of sonicated tissue volume [46]. The possible side effects caused by FUS-related exposure can also be detected by MRI. For example, FUS-induced edema has been observed as hyperintensities on T2-weighted (T2w) images, which can provide anatomical details in the brain [66]. In addition, T1 and T2 contrast agents have been conjugated to microbubbles for concurrent induction of FUS-BBB modulation and detection with corresponding MRI scans [67,68]. T2* (or T2-star)-weighted (T2*w) images are able to detect extravasation of red blood cells and hence are used for evaluating vascular damages following FUS treatment [69]. McDannold et al. used the clinically approved system (InSightec ExAblate 4000, 220 kHz) to repeatedly sonicate non-human primate (*rhesus macaques*) [70] and found hypointense signals in 28 out of 185 sonicated locations. Hypointensities were often seen in regions that associated with detecting wideband emissions (indicating inertial cavitation). Whereas, FUS-induced BBB modulation can be achieved with increased emissions at harmonics (without inertial cavitation) and result in contrast enhancement on T1w images without hypointensities on T2*w images (Figure 2). 

Several studies have demonstrated a variety of parameters that affect FUS-induced BBB modulation, such as driving frequency, peak negative pressure (PNP), burst length, pulse repetition frequency (PRF), and sonicating duration [71,72,73]. Upon acoustic exposure, oscillated microbubbles (MBs) interact with endothelial cells and thus facilitate the transcellular transportation. Three diagnostically FDA-approved MBs have been used successfully to induce BBB modulation, including Definity^®^ (Lantheus Medical Imaging, MA, USA), SonoVue^®^ (Bracco Imaging, MI, Italy), and Optison™ (GE Healthcare, WI, USA). Generally, MBs are around 1–2 μm in diameter and have a mean half-life of 1–1.3 min. McDannold et al. demonstrated BBB modulation using Optison™ (human serum albumin shell) and Definity^®^ (perflutren lipid shell) under similar acoustic exposure [74]. Although the probability of BBB modulation and acute tissue effects are similar, Optison™ produced a larger effect than Definity^®^, possibly due to the property of the shell. A similar study comparing Definity^®^ and SonoVue^®^ (phospholipid shell) was conducted by Wu et al., showing similar effects under the same exposure condition [75]. 

A wide range of ultrasound frequency has been employed to modulate BBB permeability in preclinical studies [76,77]. Due to the nature of ultrasound, increasing the frequency leads to focus distortion and skull heating, while lowering the frequency results in larger focal size. Mechanical index (MI = peak negative pressure (PNP)/$\sqrt{f}$, where f is the driving frequency of the ultrasound) is an indication of the ultrasound beam’s ability to cause cavitation-related bioeffects. The MI indicates that a higher pressure is required to modulate BBB when the higher frequency is used. McDannold et al. used a variety of frequencies with different peak negative pressures and found that MI between 0.42 to 0.50 could cause BBB modulation with minimal damages [78]. For clinical application, the ideal frequency range is between 0.2 and 1.5 MHz in hemispherically arrayed design [79] since the higher frequency would not be applicable in humans.

## 3. Preclinical Tumor Studies

One of the challenges of brain tumor therapy is heterogenous vascular permeability within tumor tissues, which hence results in an uneven drug distribution. The tumor core has more drug deposition than the peripheral area [80]. FUS has been used to increase the vascular permeability and deliver a variety of therapeutics in different types of preclinical disease models. To date, the application of FUS-mediated BBB modulation for drug delivery against tumors has been applied across many locations in the CNS such as cerebrum, brain stem, and spine [81,82,83]. This technique has been used to enhance the delivery of a wide range of chemotherapeutic agents such as doxorubicin, carboplatin, and temozolomide across the BBB/BBTB. Preclinical and clinical studies have shown that ultrasound-mediated BBB modulation is promising in animals and humans. In this section, we discuss the current progress in preclinical studies using FUS. 

The first attempt of FUS-enhanced chemo-drug delivery was using pegylated liposomal doxorubicin, which provides a longer circulating time than free-form doxorubicin, for the treatment of 9 L gliosarcoma [84,85]. The early study in healthy animals showed that MBs/FUS could achieve a significantly higher doxorubicin concentration at the sonicated brain tissues when compared to unsonicated brains. Later, a tumor study demonstrated that a single treatment produced a significantly prolonged median survival compared with either no treatment or drug alone. Another study using the same tumor model and chemotherapeutic showed that a triple treatment achieved a greater reduction of tumor growth. However, there were some adverse effects resulted from doxorubicin, such as skin toxicity, impaired activity, damage to surrounding brain tissue, and tissue loss at the tumor site [81]. Modified liposomes have also been used to improve chemotherapeutic efficacy in conjunction with MBs/FUS. Interleukin-4 (IL4) receptor-targeted liposomal doxorubicin was used to treat a human brain tumor model in nonobese diabetic-severe combined immunodeficiency (NOD-scid) mice. Both drug delivery and survival were significantly improved in animals receiving MBs/FUS and the modified chemodrug [86].

The most common systemically administered adjuvant chemotherapeutic drugs for GBM treatment are carmustine (BCNU) and Temozolomide (TMZ). BCNU (1,3-bis (2-chloroethyl)-1-nitrosourea) is an alkylating chemotherapeutic agent commonly used for brain tumors. However, it requires a high amount of drug administration to achieve the therapeutic levels in the brain, which usually caused toxicity. Treatment with MBs/FUS before BCNU administration suppressed tumor progression and prolonged animal survival compared with control [87]. TMZ is also an alkylating agent of the imidazotetrazine series that possesses strong antineoplastic activity against high-grade glioma such as recurrent GBM [88]. A series of studies using TMZ as a chemotherapeutic agent to treat brain tumors in both rodent and human tumor cell lines showed a reduction of tumor growth and an increased median survival time [89,90]. Another alkylating anticancer drug, named carboplatin, is used to treat different extracranial tumors in clinic. The use of MBs/FUS showed a promising result of enabling carboplatin delivery to the brain and yielded a therapeutic effect against a rat F98 glioma model [91]. An acoustic feedback control was employed when performing this treatment in a clinical system. Recently, another study used paclitaxel, which is a plant alkaloid, to treat an intracranial patient-derived xenograft (PDX) mouse model. With MBs/FUS enhanced delivery, an increased paclitaxel level in the brain tumor is feasible as well as an effective anti-cancer treatment [92]. Table 2 summarizes the preclinical studies combining FUS with chemo-therapeutics against brain tumors.

In addition to chemo-drug, a variety of therapeutics were used to fight malignant tumors in the brain. Firstly, a patient-specific antibody (EphA2-4B3, which is an IgG2a antibody against human EphA2-Fc immunogen) was delivered to the BBB intact PDX mouse model using MBs/FUS. Secondly, a short-hairpin RNA (shRNA), which is an artificial RNA molecule with a tight hairpin turn that can be used to silence target gene expression via RNA interference (RNAi), was delivered as an encapsulated liposome complex to suppress the progression of glioma in a rat model [93]. Moreover, multi-functional microbubbles, which are capable of targeting tumors by VEGFR2 antibody and transfecting tumors with pLUC (Luciferase reporter gene) and pHSV-TK (Herpes Simplex Virus-1 Thymidine Kinase suicide gene), were employed with FUS to inhibit tumor growth and prolong survival time [94]. Interleukin-12 (IL-12) combined with FUS-induced BBB modulation successfully triggered anticancer immune response and improved the treatment efficacy [61]. Finally, engineered natural killer cells that were delivered in brain tumors using focused ultrasound BBB modulation were found to be able to control tumors more effectively that the cells without ultrasound [95].

## 4. Clinical Trials on BBB Modulation for Drug Delivery

### 4.1. ExAblate MRgFUS System

A few clinical trials on BBB modulation for chemo-drug delivery to brain tumors using InSightec’s ExAblate MRgFUS system have been carried out in the past few years. The FUS transducer array consists of 1024 elements at a central frequency of 230 kHz. The first feasibility and safety Phase I study was performed between 2015 and 2017 at the Sunnybrook hospital in Toronto, Canada [96]. Five patients with high-grade glioma underwent the MRgFUS procedure in conjunction with administration of chemotherapy (n = 1 liposomal doxorubicin, n = 4 temozolomide) one day prior to their scheduled surgical resection. One hour prior to sonications, patients were systemically administered a sub-therapeutic dose of chemotherapy. The administration was timed so the concentration would be maximal at the expected time of BBB modulation. Targeting was based on intraoperative T2-weight MR images. Definity microbubbles was injected in boluses at a dose of 4 μL/kg per bolus immediately before initiations of sonications. A maximum of 5 locations were sonicated per patient with the total dose of Definity within 20 μL/kg, as recommended on the label for diagnostic ultrasound. At each location, acoustic beam was electronically steered over a 3-by-3 grid with 3 mm spacing to increase the coverage volume to about 9 × 9 × 6 mm^3^. A pulse train of short pulses of overall duty cycle close to 1% per sub-spot was applied [97]. Acoustic power levels varied among patients and locations from 5 to 20 W. Cavitation feedback was used as guidance for choosing the appropriate power level for a certain sonication. Samples of “sonicated” and “unsonicated” tissue were measured for the chemotherapy by liquid-chromatography-mass spectrometry. The procedure was well-tolerated, with no adverse clinical or radiologic events related to the procedure. The BBB within the target volumes showed an immediate 15–50% increase in gadolinium contrast enhancement on T1-weighted MRI, and resolution approximately 20 h after in the follow-up imaging. Dark spots in T2*-weight images indicating extravasation of red-blood cells were observed in some locations. Biochemical analysis of sonicated versus unsonicated tissue suggested that enhanced chemotherapy delivery is feasible. 

Based on the promising results, a multi-center clinical trial was initiated in 2018 on repeated chemo-drug delivery for high-grade glioma in patients undergoing standard chemotherapy. Patients were treated once per month for a maximum of 6 cycles. The Korean group first published their results of 6 patients undergoing repeated BBB modulation of a total of 33 treatments [98]. Only 4 to 6 locations were targeted per treatment due to the dose limit of Definity microbubbles, for a total of 145 locations in 6 patients. Gadolinium enhancements were observed in 131 locations (90.3%). Dark spots in T2*-weight images were observed in 93 locations (64.1%), but all resolved in follow-up MR images about a month later. There were no adverse events such as hemorrhage, edema, or neurological deficits during the entire 6-cycle period.

For ongoing trials at the Sunnybrook hospital, a higher dose of Definity microbubbles at 150 μL/kg has been approved by Health Canada to increase the treatment volume. Peripheral tumor volumes were effectively fully covered in recent cases with the increased microbubble dosage. Clinical trials on the feasibility of BBB modulation for non-cancer applications have been published for Alzheimer’s disease [99] and Amyotrophic Lateral Sclerosis (ALS) [100]. The ExAblate software has been upgraded lately to automatically modulate acoustic powers based on cavitation feedback. With recent technical advancement, the consistency of BBB modulation is expected to be improved among the patient population.

### 4.2. SonoCloud Implantable Device

The first reported clinical trial of BBB modulation used an implantable device: SonoCloud [47]. The device is 11.5 mm in diameter, which is implanted within the skull bone in the extradural space overlying the tumor area, either during a planned debulking surgical procedure or under local anesthesia in a 15-min procedure. This procedure consisted of a 3-cm skin opening, creation of a burr hole without dura matter opening, and, finally, implantation of the device and closure of the skin. 

Nineteen GBM patients were treated between 2014 and 2016 [47,101]. Patients were treated monthly for up to a maximum of six treatments or until there was evidence of tumor progression. The unfocused US transducer is 10 mm in diameter and had a center frequency of 1.05 MHz. Burst length of 23.8 ms at 1 Hz PRF was applied for 150 s. Acoustic pressure levels between 0.41 and 1.15 MPa were tested over the patient population with systemic infusion of carboplatin for 60 to 90 min following the BBB modulation procedure. SonoVue microbubbles were delivered in bolus at 0.1 mL/kg. No treatment-related serious adverse events were reported. BBB disruption was observed in 52 of 65 treatments at acoustic pressure of 0.8 MPa or higher based on Gadolinium enhancement in T1-weighted MR images. 

Once implanted, the device has the advantage of convenience for repeated sonications. The disadvantages, on the other hand, are limited coverage volume and limited control of acoustic pressure over the target volume. Fixed acoustic pressure was applied without cavitation feedback. The InSightec transcranial system and the SonoCloud implantable device may work best for different applications and patient population.

### 4.3. NaviFUS Neuronavigation-Guided System

A neuronavigation-guided FUS system, NaviFUS, will be used in a phase I clinical trial in chemo-drug (bevacizumab) delivery for recurrent glioblastoma patients [102]. The system consists of a FUS array in conjunction with a clinically available neuronavigation system. The patient will be fixed semi-rigidly without a headpin system. Burst length of 10 ms at PRF of 9 Hz will be applied to a 3 × 3 grid with 5 mm spacing. Various acoustic pressure levels will be tested with bolus injections of SonoVue microbbubles at 0.1 mL/kg. A center frequency of 500 kHz will be used with a real-time acoustic emission/reflection monitoring. It remains to be seen if the neuronavigation-guided approach will provide an accurate yet convenient way for repeated BBB modulations in clinical practice.

## 5. Conclusions

Emerging approaches bypassing the barrier have been developed to treat brain tumors. FUS provides one of the most potential progresses achieving non-invasive and reproducible drug delivery against the malignant tumor in the brain. The efficacy has been shown not only in preclinical scales but also in clinical trials. Although it is in the early investigational stage until this technology can be fully used in clinic, it has the potential to alter the precision medicine in treating brain tumor patients.

## Figures and Tables

**Table 1:** Summary of strategies overcoming blood–brain barrier (BBB)/blood–brain–tumor barrier (BBTB).

Methods	Advantages	Disadvantages
Direct surgical injection	Local delivery via bulk flow	Highly invasive; poor distribution
Intranasal delivery	Non-invasive; rapid absorption	Only suitable for potent drugs (undegraded via mucosal enzymes)
AET-targeted strategies	Non-invasive; highly specific	Insufficient efficacy and intolerable toxicity
TJ-targeted strategies	Reversible	Non-selective permeabilization and invasive injection required
Focused ultrasound-induced brain vascular permeability increment	Non-invasive; localized; transient; reversible	Precise controls need further exploration; sophisticated equipment required

**Table 2:** Representative animal studies using FUS and therapeutics across the BBB/BBTB.

Therapeutic Agent	Animal Model	Outcome	References
Liposomal doxorubicin	Rat 9 L gliosarcoma	Increased median survival time (*p* = 0.0007)	Treat et al. [85]
IL4-liposomal doxorubicin	Human GBM 8401	Improved median survival time (*p* = 0.0001)	Yang et al. [86]
1,3-bis(2-chloroethyl)-1-nitrosourea (BCNU)	Rat C6 glioma	Improved median survival time (*p* = 0.015)	Liu et al. [87]
Temozolomide	Rat 9L gliosarcoma	Mean survival time increased 37.7%	Wei et al. [89]
Temozolomide	Human U87 glioma	Prolonged mean survival time (*p* < 0.05)	Liu et al. [90]
Carboplatin	Rat F98 glioma	Increase median survival time (*p* < 0.01)	McDannold et al. [91]
Paclitaxel	Patient-derived glioma xenografts (PDX)	In MES83 model: extended median survival (*p* = 0.0006); in GBM12 model, increased survival (*p* < 0.001)	Zhang et al. [92]

## References

[1] Di Giovanna A.P., Tibo A., Silvestri L., Mullenbroich M.C., Costantini I., Allegra Mascaro A.L., Sacconi L., Frasconi P., Pavone F.S. (2018). Whole-Brain Vasculature Reconstruction at the Single Capillary Level. Sci. Rep..

[2] van Tellingen O., Yetkin-Arik B., de Gooijer M.C., Wesseling P., Wurdinger T., de Vries H.E. (2015). Overcoming the blood-brain tumor barrier for effective glioblastoma treatment. Drug Resist. Updates.

[3] Hansen A.J. (1985). Effect of anoxia on ion distribution in the brain. Physiol. Rev..

[4] Abbott N.J., Ronnback L., Hansson E. (2006). Astrocyte-endothelial interactions at the blood-brain barrier. Nat. Rev. Neurosci..

[5] Zahraoui A., Louvard D., Galli T. (2000). Tight junction, a platform for trafficking and signaling protein complexes. J. Cell Biol..

[6] Zlokovic B.V. (2008). The blood-brain barrier in health and chronic neurodegenerative disorders. Neuron.

[7] Daneman R., Prat A. (2015). The blood-brain barrier. Cold Spring Harb. Perspect. Biol..

[8] Ha S.N., Hochman J., Sheridan R.P. (2007). Mini review on molecular modeling of P-glycoprotein (Pgp). Curr. Top. Med. Chem..

[9] Ambudkar S.V., Lelong I.H., Zhang J., Cardarelli C.O., Gottesman M.M., Pastan I. (1992). Partial purification and reconstitution of the human multidrug-resistance pump: Characterization of the drug-stimulatable ATP hydrolysis. Proc. Natl. Acad. Sci. USA.

[10] Obermeier B., Daneman R., Ransohoff R.M. (2013). Development, maintenance and disruption of the blood-brain barrier. Nat. Med..

[11] Dubois L.G., Campanati L., Righy C., D’Andrea-Meira I., Spohr T.C., Porto-Carreiro I., Pereira C.M., Balca-Silva J., Kahn S.A., DosSantos M.F. (2014). Gliomas and the vascular fragility of the blood brain barrier. Front. Cell. Neurosci..

[12] Watkins S., Robel S., Kimbrough I.F., Robert S.M., Ellis-Davies G., Sontheimer H. (2014). Disruption of astrocyte-vascular coupling and the blood-brain barrier by invading glioma cells. Nat. Commun..

[13] Machein M.R., Kullmer J., Fiebich B.L., Plate K.H., Warnke P.C. (1999). Vascular endothelial growth factor expression, vascular volume, and, capillary permeability in human brain tumors. Neurosurgery.

[14] Hardee M.E., Zagzag D. (2012). Mechanisms of glioma-associated neovascularization. Am. J. Pathol..

[15] Plate K.H., Scholz A., Dumont D.J. (2012). Tumor angiogenesis and anti-angiogenic therapy in malignant gliomas revisited. Acta Neuropathol..

[16] Cao Y., Sundgren P.C., Tsien C.I., Chenevert T.T., Junck L. (2006). Physiologic and metabolic magnetic resonance imaging in gliomas. J. Clin. Oncol..

[17] Wang D., Wang C., Wang L., Chen Y. (2019). A comprehensive review in improving delivery of small-molecule chemotherapeutic agents overcoming the blood-brain/brain tumor barriers for glioblastoma treatment. Drug Deliv..

[18] Allhenn D., Boushehri M.A., Lamprecht A. (2012). Drug delivery strategies for the treatment of malignant gliomas. Int. J. Pharm..

[19] Jahangiri A., Chin A.T., Flanigan P.M., Chen R., Bankiewicz K., Aghi M.K. (2017). Convection-enhanced delivery in glioblastoma: A review of preclinical and clinical studies. J. Neurosurg..

[20] Yang W., Barth R.F., Huo T., Nakkula R.J., Weldon M., Gupta N., Agius L., Grecula J.C. (2014). Radiation therapy combined with intracerebral administration of carboplatin for the treatment of brain tumors. Radiat. Oncol..

[21] Lidar Z., Mardor Y., Jonas T., Pfeffer R., Faibel M., Nass D., Hadani M., Ram Z. (2004). Convection-enhanced delivery of paclitaxel for the treatment of recurrent malignant glioma: A phase I/II clinical study. J. Neurosurg..

[22] Mueller S., Polley M.Y., Lee B., Kunwar S., Pedain C., Wembacher-Schroder E., Mittermeyer S., Westphal M., Sampson J.H., Vogelbaum M.A. (2011). Effect of imaging and catheter characteristics on clinical outcome for patients in the PRECISE study. J. Neurooncol..

[23] Westphal M., Hilt D.C., Bortey E., Delavault P., Olivares R., Warnke P.C., Whittle I.R., Jaaskelainen J., Ram Z. (2003). A phase 3 trial of local chemotherapy with biodegradable carmustine (BCNU) wafers (Gliadel wafers) in patients with primary malignant glioma. Neuro-Oncology.

[24] Chowdhary S.A., Ryken T., Newton H.B. (2015). Survival outcomes and safety of carmustine wafers in the treatment of high-grade gliomas: A meta-analysis. J. Neurooncol..

[25] Bregy A., Shah A.H., Diaz M.V., Pierce H.E., Ames P.L., Diaz D., Komotar R.J. (2013). The role of Gliadel wafers in the treatment of high-grade gliomas. Expert Rev. Anticancer Ther..

[26] Masi B.C., Tyler B.M., Bow H., Wicks R.T., Xue Y., Brem H., Langer R., Cima M.J. (2012). Intracranial MEMS based temozolomide delivery in a 9L rat gliosarcoma model. Biomaterials.

[27] Bastiancich C., Vanvarenberg K., Ucakar B., Pitorre M., Bastiat G., Lagarce F., Preat V., Danhier F. (2016). Lauroyl-gemcitabine-loaded lipid nanocapsule hydrogel for the treatment of glioblastoma. J. Control. Release.

[28] Bidros D.S., Vogelbaum M.A. (2009). Novel drug delivery strategies in neuro-oncology. Neurotherapeutics.

[29] Kunwar S., Chang S., Westphal M., Vogelbaum M., Sampson J., Barnett G., Shaffrey M., Ram Z., Piepmeier J., Prados M. (2010). Phase III randomized trial of CED of IL13-PE38QQR vs Gliadel wafers for recurrent glioblastoma. Neuro-Oncology.

[30] Bruinsmann F.A., Richter Vaz G., de Cristo Soares Alves A., Aguirre T., Raffin Pohlmann A., Stanisçuaski Guterres S., Sonvico F. (2019). Nasal Drug Delivery of Anticancer Drugs for the Treatment of Glioblastoma: Preclinical and Clinical Trials. Molecules.

[31] da Fonseca C.O., Khandelia H., Salazar M.D., Schönthal A.H., Meireles O.C., Quirico-Santos T. (2016). Perillyl alcohol: Dynamic interactions with the lipid bilayer and implications for long-term inhalational chemotherapy for gliomas. Surg. Neurol. Int..

[32] da Fonseca C.O., Schwartsmann G., Fischer J., Nagel J., Futuro D., Quirico-Santos T., Gattass C.R. (2008). Preliminary results from a phase I/II study of perillyl alcohol intranasal administration in adults with recurrent malignant gliomas. Surg. Neurol..

[33] da Fonseca C.O., Simão M., Lins I.R., Caetano R.O., Futuro D., Quirico-Santos T. (2011). Efficacy of monoterpene perillyl alcohol upon survival rate of patients with recurrent glioblastoma. J. Cancer Res. Clin. Oncol..

[34] Bradley M.O., Webb N.L., Anthony F.H., Devanesan P., Witman P.A., Hemamalini S., Chander M.C., Baker S.D., He L., Horwitz S.B. (2001). Tumor targeting by covalent conjugation of a natural fatty acid to paclitaxel. Clin. Cancer Res..

[35] Gaillard P.J., Appeldoorn C.C., Dorland R., van Kregten J., Manca F., Vugts D.J., Windhorst B., van Dongen G.A., de Vries H.E., Maussang D. (2014). Pharmacokinetics, brain delivery, and efficacy in brain tumor-bearing mice of glutathione pegylated liposomal doxorubicin (2B3-101). PLoS ONE.

[36] Oberoi R.K., Parrish K.E., Sio T.T., Mittapalli R.K., Elmquist W.F., Sarkaria J.N. (2016). Strategies to improve delivery of anticancer drugs across the blood-brain barrier to treat glioblastoma. Neuro-Oncology.

[37] Bankstahl J.P., Bankstahl M., Romermann K., Wanek T., Stanek J., Windhorst A.D., Fedrowitz M., Erker T., Muller M., Loscher W. (2013). Tariquidar and elacridar are dose-dependently transported by P-glycoprotein and Bcrp at the blood-brain barrier: A small-animal positron emission tomography and in vitro study. Drug Metab. Dispos..

[38] Kurnik D., Sofowora G.G., Donahue J.P., Nair U.B., Wilkinson G.R., Wood A.J., Muszkat M. (2008). Tariquidar, a selective P-glycoprotein inhibitor, does not potentiate loperamide’s opioid brain effects in humans despite full inhibition of lymphocyte P-glycoprotein. Anesthesiology.

[39] Bhowmik A., Khan R., Ghosh M.K. (2015). Blood brain barrier: A challenge for effectual therapy of brain tumors. Biomed. Res. Int..

[40] Siegal T., Rubinstein R., Bokstein F., Schwartz A., Lossos A., Shalom E., Chisin R., Gomori J.M. (2000). In vivo assessment of the window of barrier opening after osmotic blood-brain barrier disruption in humans. J. Neurosurg..

[41] Rodriguez A., Tatter S.B., Debinski W. (2015). Neurosurgical Techniques for Disruption of the Blood-Brain Barrier for Glioblastoma Treatment. Pharmaceutics.

[42] Gerber N.U., Mynarek M., von Hoff K., Friedrich C., Resch A., Rutkowski S. (2014). Recent developments and current concepts in medulloblastoma. Cancer Treat. Rev..

[43] Kemper E.M., Boogerd W., Thuis I., Beijnen J.H., van Tellingen O. (2004). Modulation of the blood-brain barrier in oncology: Therapeutic opportunities for the treatment of brain tumours?. Cancer Treat. Rev..

[44] Pardridge W.M. (2005). The blood-brain barrier and neurotherapeutics. NeuroRX.

[45] Hynynen K., McDannold N., Vykhodtseva N., Jolesz F.A. (2001). Noninvasive MR imaging-guided focal opening of the blood-brain barrier in rabbits. Radiology.

[46] O’Reilly M.A., Hough O., Hynynen K. (2017). Blood-Brain Barrier Closure Time After Controlled Ultrasound-Induced Opening Is Independent of Opening Volume. J. Ultrasound Med..

[47] Carpentier A., Canney M., Vignot A., Reina V., Beccaria K., Horodyckid C., Karachi C., Leclercq D., Lafon C., Chapelon J.-Y. (2016). Clinical trial of blood-brain barrier disruption by pulsed ultrasound. Sci. Transl. Med..

[48] McDannold N., Vykhodtseva N., Hynynen K. (2006). Targeted disruption of the blood-brain barrier with focused ultrasound: Association with cavitation activity. Phys. Med. Biol..

[49] Liu H.-L., Wai Y.-Y., Chen W.-S., Chen J.-C., Hsu P.-H., Wu X.-Y., Huang W.-C., Yen T.-C., Wang J.-J. (2008). Hemorrhage detection during focused-ultrasound induced blood-brain-barrier opening by using susceptibility-weighted magnetic resonance imaging. Ultrasound Med. Biol..

[50] Bader K.B., Holland C.K. (2013). Gauging the likelihood of stable cavitation from ultrasound contrast agents. Phys. Med. Biol..

[51] O’Reilly M.A., Hynynen K. (2012). Blood-brain barrier: Real-time feedback-controlled focused ultrasound disruption by using an acoustic emissions-based controller. Radiology.

[52] Burgess A., Shah K., Hough O., Hynynen K. (2015). Focused ultrasound-mediated drug delivery through the blood-brain barrier. Expert Rev. Neurother..

[53] Sheikov N., McDannold N., Vykhodtseva N., Jolesz F., Hynynen K. (2004). Cellular mechanisms of the blood-brain barrier opening induced by ultrasound in presence of microbubbles. Ultrasound Med. Biol..

[54] Liu H.-L., Hsu P.-H., Chu P.-C., Wai Y.-Y., Chen J.-C., Shen C.-R., Yen T.-C., Wang J.-J. (2009). Magnetic resonance imaging enhanced by superparamagnetic iron oxide particles: Usefulness for distinguishing between focused ultrasound-induced blood-brain barrier disruption and brain hemorrhage. J. Magn. Reson. Imaging.

[55] Cherry J.D., Olschowka J.A., O’Banion M.K. (2014). Neuroinflammation and M2 microglia: The good, the bad, and the inflamed. J. Neuroinflammation.

[56] McMahon D., Hynynen K. (2017). Acute Inflammatory Response Following Increased Blood-Brain Barrier Permeability Induced by Focused Ultrasound is Dependent on Microbubble Dose. Theranostics.

[57] Kovacs Z.I., Kim S., Jikaria N., Qureshi F., Milo B., Lewis B.K., Bresler M., Burks S.R., Frank J.A. (2017). Disrupting the blood–brain barrier by focused ultrasound induces sterile inflammation. Proc. Natl. Acad. Sci. USA.

[58] Silburt J., Lipsman N., Aubert I. (2017). Disrupting the blood–brain barrier with focused ultrasound: Perspectives on inflammation and regeneration. Proc. Natl. Acad. Sci. USA.

[59] Kovacs Z.I., Burks S.R., Frank J.A. (2018). Focused ultrasound with microbubbles induces sterile inflammatory response proportional to the blood brain barrier opening: Attention to experimental conditions. Theranostics.

[60] McMahon D., Bendayan R., Hynynen K. (2017). Acute effects of focused ultrasound-induced increases in blood-brain barrier permeability on rat microvascular transcriptome. Sci. Rep..

[61] Chen P.-Y., Hsieh H.-Y., Huang C.-Y., Lin C.-Y., Wei K.-C., Liu H.-L. (2015). Focused ultrasound-induced blood-brain barrier opening to enhance interleukin-12 delivery for brain tumor immunotherapy: A preclinical feasibility study. J. Transl. Med..

[62] McMahon D., Mah E., Hynynen K. (2018). Angiogenic response of rat hippocampal vasculature to focused ultrasound-mediated increases in blood-brain barrier permeability. Sci. Rep..

[63] Park J., Zhang Y., Vykhodtseva N., Jolesz F.A., McDannold N.J. (2012). The kinetics of blood brain barrier permeability and targeted doxorubicin delivery into brain induced by focused ultrasound. J. Control. Release.

[64] Chai W.Y., Chu P.C., Tsai M.Y., Lin Y.C., Wang J.J., Wei K.C., Wai Y.Y., Liu H.L. (2014). Magnetic-resonance imaging for kinetic analysis of permeability changes during focused ultrasound-induced blood-brain barrier opening and brain drug delivery. J. Control. Release.

[65] Chai W.Y., Chu P.C., Tsai C.H., Lin C.Y., Yang H.W., Lai H.Y., Liu H.L. (2018). Image-Guided Focused-Ultrasound CNS Molecular Delivery: An Implementation via Dynamic Contrast-Enhanced Magnetic-Resonance Imaging. Sci. Rep..

[66] Downs M.E., Buch A., Karakatsani M.E., Konofagou E.E., Ferrera V.P. (2015). Blood-Brain Barrier Opening in Behaving Non-Human Primates via Focused Ultrasound with Systemically Administered Microbubbles. Sci. Rep..

[67] Liao A.-H., Liu H.-L., Su C.-H., Hua M.-Y., Yang H.-W., Weng Y.-T., Hsu P.-H., Huang S.-M., Wu S.-Y., Wang H.-E. (2012). Paramagnetic perfluorocarbon-filled albumin-(Gd-DTPA) microbubbles for the induction of focused-ultrasound-induced blood-brain barrier opening and concurrent MR and ultrasound imaging. Phys. Med. Biol..

[68] Fan C.-H., Ting C.-Y., Lin H.-J., Wang C.-H., Liu H.-L., Yen T.-C., Yeh C.-K. (2013). SPIO-conjugated, doxorubicin-loaded microbubbles for concurrent MRI and focused-ultrasound enhanced brain-tumor drug delivery. Biomaterials.

[69] Tsushima Y., Endo K. (2006). Hypointensities in the brain on T2*-weighted gradient-echo magnetic resonance imaging. Curr. Probl. Diagn. Radiol..

[70] McDannold N., Arvanitis C.D., Vykhodtseva N., Livingstone M.S. (2012). Temporary disruption of the blood-brain barrier by use of ultrasound and microbubbles: Safety and efficacy evaluation in rhesus macaques. Cancer Res..

[71] Hynynen K., McDannold N., Sheikov N.A., Jolesz F.A., Vykhodtseva N. (2005). Local and reversible blood-brain barrier disruption by noninvasive focused ultrasound at frequencies suitable for trans-skull sonications. Neuroimage.

[72] McDannold N., Vykhodtseva N., Hynynen K. (2008). Effects of acoustic parameters and ultrasound contrast agent dose on focused-ultrasound induced blood-brain barrier disruption. Ultrasound Med. Biol.

[73] O’Reilly M.A., Waspe A.C., Ganguly M., Hynynen K. (2011). Focused-ultrasound disruption of the blood-brain barrier using closely-timed short pulses: Influence of sonication parameters and injection rate. Ultrasound Med. Biol..

[74] McDannold N., Vykhodtseva N., Hynynen K. (2007). Use of Ultrasound Pulses Combined with Definity for Targeted Blood-Brain Barrier Disruption: A Feasibility Study. Ultrasound Med. Biol..

[75] Wu S.K., Chu P.C., Chai W.Y., Kang S.T., Tsai C.H., Fan C.H., Yeh C.K., Liu H.L. (2017). Characterization of different microbubbles in assisting focused ultrasound-induced blood-brain barrier opening. Sci. Rep..

[76] Liu H.L., Pan C.H., Ting C.Y., Hsiao M.J. (2010). Opening of the Blood-Brain Barrier by Low-Frequency (28-kHz) Ultrasound: A Novel Pinhole-Assisted Mechanical Scanning Device. Ultrasound Med. Biol..

[77] Bing K.F., Howles G.P., Qi Y., Palmeri M.L., Nightingale K.R. (2009). Blood-Brain Barrier (BBB) Disruption Using a Diagnostic Ultrasound Scanner and Definity^®^ in Mice. Ultrasound Med. Biol..

[78] McDannold N., Vykhodtseva N., Hynynen K. (2008). Blood-Brain Barrier Disruption Induced by Focused Ultrasound and Circulating Preformed Microbubbles Appears to Be Characterized by the Mechanical Index. Ultrasound Med. Biol..

[79] Pajek D., Hynynen K. (2013). The application of sparse arrays in high frequency transcranial focused ultrasound therapy: A simulation study. Med. Phys..

[80] Ewing J.R., Brown S.L., Lu M., Panda S., Ding G., Knight R.A., Cao Y., Jiang Q., Nagaraja T.N., Churchman J.L. (2006). Model selection in magnetic resonance imaging measurements of vascular permeability: Gadomer in a 9L model of rat cerebral tumor. J. Cereb. Blood Flow Metab..

[81] Aryal M., Vykhodtseva N., Zhang Y.-Z., Park J., McDannold N. (2013). Multiple treatments with liposomal doxorubicin and ultrasound-induced disruption of blood-tumor and blood-brain barriers improve outcomes in a rat glioma model. J. Control. Release.

[82] O’Reilly M.A., Chinnery T., Yee M.L., Wu S.K., Hynynen K., Kerbel R.S., Czarnota G.J., Pritchard K.I., Sahgal A. (2018). Preliminary Investigation of Focused Ultrasound-Facilitated Drug Delivery for the Treatment of Leptomeningeal Metastases. Sci. Rep..

[83] Alli S., Figueiredo C.A., Golbourn B., Sabha N., Wu M.Y., Bondoc A., Luck A., Coluccia D., Maslink C., Smith C. (2018). Brainstem blood brain barrier disruption using focused ultrasound: A demonstration of feasibility and enhanced doxorubicin delivery. J. Control. Release.

[84] Treat L.H., McDannold N., Vykhodtseva N., Zhang Y., Tam K., Hynynen K. (2007). Targeted delivery of doxorubicin to the rat brain at therapeutic levels using MRI-guided focused ultrasound. Int. J. Cancer.

[85] Treat L.H., McDannold N., Zhang Y., Vykhodtseva N., Hynynen K. (2012). Improved anti-tumor effect of liposomal doxorubicin after targeted blood-brain barrier disruption by MRI-guided focused ultrasound in rat glioma. Ultrasound Med. Biol..

[86] Yang F.-Y., Wong T.-T., Teng M.-C., Liu R.-S., Lu M., Liang H.-F., Wei M.-C. (2012). Focused ultrasound and interleukin-4 receptor-targeted liposomal doxorubicin for enhanced targeted drug delivery and antitumor effect in glioblastoma multiforme. J. Control. Release.

[87] Liu H.-L., Hua M.-Y., Chen P.-Y., Chu P.-C., Pan C.-H., Yang H.-W., Huang C.-Y., Wang J.-J., Yen T.-C., Wei K.-C. (2010). Blood-brain barrier disruption with focused ultrasound enhances delivery of chemotherapeutic drugs for glioblastoma treatment. Radiology.

[88] Kim H.K., Lin C.C., Parker D., Veals J., Lim J., Likhari P., Statkevich P., Marco A., Nomeir A.A. (1997). High-performance liquid chromatographic determination and stability of 5-(3-methyltriazen-1-yl)-imidazo-4-carboximide, the biologically active product of the antitumor agent temozolomide, in human plasma. J. Chromatogr. B Biomed. Sci. Appl..

[89] Wei K.C., Chu P.C., Wang H.Y., Huang C.Y., Chen P.Y., Tsai H.C., Lu Y.J., Lee P.Y., Tseng I.C., Feng L.Y. (2013). Focused ultrasound-induced blood-brain barrier opening to enhance temozolomide delivery for glioblastoma treatment: A preclinical study. PLoS ONE.

[90] Liu H.-L.L., Huang C.-Y.Y., Chen J.Y., Wang H.Y., Chen P.-Y.Y., Wei K.-C.C., Treat L.H., McDannold N., Zhang Y.-Z., Vykhodtseva N. (2014). Pharmacodynamic and therapeutic investigation of focused ultrasound-induced blood-brain barrier opening for enhanced temozolomide delivery in glioma treatment. PLoS ONE.

[91] McDannold N., Zhang Y., Supko J.G., Power C., Sun T., Peng C., Vykhodtseva N., Golby A.J., Reardon D.A. (2019). Acoustic feedback enables safe and reliable carboplatin delivery across the blood-brain barrier with a clinical focused ultrasound system and improves survival in a rat glioma model. Theranostics.

[92] Zhang D.Y., Dmello C., Chen L., Arrieta V.A., Gonzalez-Buendia E., Kane J.R., Magnusson L.P., Baran A., James C.D., Horbinski C. (2020). Ultrasound-mediated Delivery of Paclitaxel for Glioma: A Comparative Study of Distribution, Toxicity, and Efficacy of Albumin-bound Versus Cremophor Formulations. Clin. Cancer Res..

[93] Zhao G., Huang Q., Wang F., Zhang X., Hu J., Tan Y., Huang N., Wang Z., Wang Z., Cheng Y. (2018). Targeted shRNA-loaded liposome complex combined with focused ultrasound for blood brain barrier disruption and suppressing glioma growth. Cancer Lett..

[94] Chang E.-L., Ting C.-Y., Hsu P.-H., Lin Y.-C., Liao E.-C., Huang C.-Y., Chang Y.-C., Chan H.-L., Chiang C.-S., Liu H.-L. (2017). Angiogenesis-targeting microbubbles combined with ultrasound-mediated gene therapy in brain tumors. J. Control. Release.

[95] Alkins R., Burgess A., Kerbel R., Wels W.S., Hynynen K. (2016). Early treatment of HER2-amplified brain tumors with targeted NK-92 cells and focused ultrasound improves survival. Neuro-Oncology.

[96] Mainprize T., Lipsman N., Huang Y., Meng Y., Bethune A., Ironside S., Heyn C., Alkins R., Trudeau M., Sahgal A. (2019). Blood-Brain Barrier Opening in Primary Brain Tumors with Non-invasive MR-Guided Focused Ultrasound: A Clinical Safety and Feasibility Study. Sci. Rep..

[97] Huang Y., Alkins R., Schwartz M.L., Hynynen K. (2017). Opening the blood-brain barrier with MR imaging-guided focused ultrasound: Preclinical testing on a trans-human skull porcine model. Radiology.

[98] Park S.H., Kim M.J., Jung H.H., Chang W.S., Choi H.S., Rachmilevitch I., Zadicario E., Chang J.W. (2020). Safety and feasibility of multiple blood-brain barrier disruptions for the treatment of glioblastoma in patients undergoing standard adjuvant chemotherapy. J. Neurosurg..

[99] Lipsman N., Meng Y., Bethune A.J., Huang Y., Lam B., Masellis M., Herrmann N., Heyn C., Aubert I., Boutet A. (2018). Blood–brain barrier opening in Alzheimer’s disease using MR-guided focused ultrasound. Nat. Commun..

[100] Abrahao A., Meng Y., Llinas M., Huang Y., Hamani C., Mainprize T., Aubert I., Heyn C., Black S.E., Hynynen K. (2019). First-in-human trial of blood–brain barrier opening in amyotrophic lateral sclerosis using MR-guided focused ultrasound. Nat. Commun..

[101] Idbaih A., Canney M., Belin L., Desseaux C., Vignot A., Bouchoux G., Asquier N., Law-Ye B., Leclercq D., Bissery A. (2019). Safety and feasibility of repeated and transient blood-brain barrier disruption by pulsed ultrasound in patients with recurrent glioblastoma. Clin. Cancer Res..

[102] Chen K.-T., Lin Y.-J., Chai W.-Y., Lin C.-J., Chen P.-Y., Huang C.-Y., Kuo J.S., Liu H.-L., Wei K.-C. (2020). Neuronavigation-guided focused ultrasound (NaviFUS) for transcranial blood-brain barrier opening in recurrent glioblastoma patients: Clinical trial protocol. Ann. Transl. Med..

